# Natriuretic Peptides in the Management of Solid Organ Transplantation Associated Acute Kidney Injury: A Systematic Review and Meta-Analysis

**DOI:** 10.1155/2013/949357

**Published:** 2013-05-16

**Authors:** Sagar U. Nigwekar, Hrishikesh Kulkarni, Charuhas V. Thakar

**Affiliations:** ^1^Division of Nephrology, Massachusetts General Hospital, Bulfinch 127, Boston, MA 02114, USA; ^2^Scholars in Clinical Science Program, Harvard Medical School, Boston, MA 02115, USA; ^3^Department of Medicine, University of Pittsburgh School of Medicine, Pittsburgh, PA 15213, USA; ^4^Division of Nephrology, University of Cincinnati, Cincinnati, OH 45220, USA; ^5^Cincinnati VA Medical Center, Cincinnati, OH 45220, USA

## Abstract

Randomized controlled trials involving natriuretic peptide administration in solid organ transplantation setting have shown inconsistent effects for renal endpoints. We conducted a systematic review and meta-analysis of these trials to ascertain the role of natriuretic peptides in the management of solid organ transplantation associated acute kidney injury (AKI). MEDLINE, EMBASE, and Google scholar were searched independently by two authors for randomized trials evaluating renal effects of natriuretic peptides in solid organ transplantation settings. Two reviewers independently assessed the studies for eligibility and extracted the relevant data. The pooled estimate showed that natriuretic peptide administration is associated with a reduction in AKI requiring dialysis (odds ratio = 0.50 [0.26–0.97]), a statistically nonsignificant trend toward improvement in posttransplant creatinine clearance (weighted mean difference = 5.5 mL/min, [−1.3 to 12.2 mL/min]), and reduction in renal replacement requirement duration (weighted mean difference −44.0 hours, [−60.5 to −27.5 hours]). There were no mortality events and no adverse events related to natriuretic peptides. In conclusion, administration of natriuretic peptides in solid organ transplantation may be associated with significant improvements in renal outcomes. These observations need to be confirmed in an adequately powered, prospective multicenter study.

## 1. Introduction

Acute kidney injury (AKI) is common in hospitalized patients and is associated with significant morbidity and mortality [1, 2]. Despite recent advances, outcomes from AKI have not substantially changed in the last four decades and the incidence of AKI is on the rise [3]. Solid organ transplantation procedures (e.g., liver transplantation, heart transplantation, lung transplantation, and combined solid organ transplantations such as heart-lung transplant) are a recognized cause of AKI and renal transplantation is also frequently associated with AKI [4–10]. The incidence of AKI after liver transplantation reportedly ranges from 12% to 67% depending upon the definition used [4, 11]. Dialysis is required in up to 21% of the cases [4], and AKI in this setting is associated with higher mortality [4, 11]. Similarly, the incidence of AKI remains high in immediate postcardiac transplantation setting as up to 1/3rd of patients develop AKI [7]. Postischemic acute tubular necrosis is the most common cause of persistent renal failure (also known as delayed graft function) in the immediate postrenal transplant period and remains a major obstacle for renal graft survival [12]. There remains an unmet need to explore novel therapeutic agents and revisit some older agents to explore their role in management of AKI in solid organ transplantation setting.

Natriuretic peptides are a family of peptides predominantly synthesized in the atrial myocyte and then stored as three different prohormones: 126-amino acid atrial natriuretic peptide prohormone, 108-amino acid brain natriuretic peptide prohormone, and 126-amino acid C-natriuretic peptide prohormone [13–17]. Posttranslational modification of atrial natriuretic peptide prohormone in the heart produces atrial natriuretic peptide, which is a 28-amino acid peptide with direct diuretic and natriuretic effects in both animals and humans [13–16]. Atrial natriuretic peptide has been shown to block tubular reabsorption of sodium promoting natriuresis, reverse endothelin-induced vasoconstriction leading to dilation of afferent arterioles, and inhibit renin-angiotensin system [14–16, 18–20]. Post-translational modification of atrial natriuretic peptide prohormone in the kidney produces urodilatin with additional four amino acids at the N-terminal [13–16]. Brain natriuretic peptide, a 32-amino acid peptide, derived from brain natriuretic peptide prohormone, has remarkable sequence homology to atrial natriuretic peptide with only four amino acids being different in the amino acid ring structure common to both peptides [13–16]. Brain natriuretic peptide also has diuretic, natriuretic, vasodilatory, and aldosterone inhibiting properties [21]. C-natriuretic peptide, derived from C-natriuretic peptide prohormone, despite having similar amino acid sequence as atrial natriuretic peptide lacks any physiological effects on intrarenal sodium handling, sodium excretion, aldosterone pathway, and hemodynamics [13–16].

Despite the above described physiologic actions and potential to reverse multiple factors involved in the pathogenesis of solid organ transplantation associated AKI (including renal ischemia and hyperactivated renin-angiotensin-aldosterone system), randomized controlled trials (RCTs) evaluating the role of natriuretic peptides in this setting have been largely underpowered and have produced conflicting results [4, 6, 22–26]. In addition, natriuretic peptides, especially at high doses, are known to cause hypotension and arrhythmias, complications that can potentially negate the possible benefits [14–17, 27]. The purpose of this review was to undertake a systematic analysis of randomized controlled studies to ascertain the therapeutic potential of natriuretic peptides in the management of AKI that occurs after solid organ transplantation procedures.

## 2. Methods

### 2.1. Data Sources, Search Strategy, and Study Selection

We performed this review as per the QUOROM statement [28]. Two reviewers searched MEDLINE (1966 to August 2012), EMBASE (1980 to August 2012), and Google scholar (in August 2012) for randomized controlled studies that compared any form or dose of natriuretic peptide to placebo or standard treatment (such as hydration and diuretics) in adult (age >18 years) patients undergoing solid organ transplantation surgery. To be included the studies had to report at least one of the prespecified renal outcomes—AKI requiring dialysis, postsurgery serum creatinine, or creatinine clearance levels. To retrieve the eligible studies, we employed the following search terms: natriuretic peptides, atrial natriuretic peptide, ANP, urodilatin, anaritide, uraliritide, atriopeptin, brain natriuretic peptide, BNP, C-type natriuretic peptide, surgery, operation, transplantation, organ transplantation, acute renal failure, acute kidney failure, ARF, acute renal insufficiency, acute kidney insufficiency, acute kidney injury, AKI, acute tubular necrosis, ATN, and delayed graft function.In addition, we studied reference lists and bibliographical data from all retrieved articles and reviews for any additional relevant material. There was no language restriction. 

Following studies were excluded: (1) nonrandomized trials, (2) those evaluating the role of natriuretic peptides in nontransplant surgical setting (e.g., cardiovascular surgeries and radiocontrast nephropathy prevention), (4) experimental animal studies, and (5) those that did not report the pre-specified renal outcomes.

### 2.2. Data Extraction and Quality Assessment

Two reviewers independently assessed the studies for eligibility and extracted relevant data regarding study design and setting, participant characteristics, and outcome measures using a standardized data extraction form (SN and HK). There were no disagreements between the 2 independent reviewers for the extracted data. Only explicit descriptions of outcome events were tabulated. If the required data could not be obtained from the journal publication, then 2 separate attempts at contacting original authors were made.

The results of the individual studies were reported in many different ways, including mean and standard deviation (SD), standard error of the mean (SEM), or interquartile range (IQR). We converted standard error of the means and interquartile ranges to standard deviation, using appropriate formulae. We considered interquartile range to be 1.35 times the standard deviation. Standard deviation was calculated as square root of sample size multiplied by the standard error of the mean. All data was converted to uniform measurements; thus serum creatinine is presented as mg/dL and creatinine clearance or glomerular filtration rate as mL/min.

The method of all included studies was rated by means of the validated scale by Jadad et al. [29]. This scale considers randomization, blinding, and withdrawal/dropouts. Studies were considered to be of low quality if the Jadad score was from 0 to 2, of moderate quality if the score was from 3 to 4, and of high quality if the score was 5. Study quality was appraised by two reviewers independently and divergences resolved by consensus. 

## 3. Outcome Measures

The primary outcomes of interest for the current review were posttransplantation AKI requiring dialysis and short term mortality (30 day or in hospital). Secondary outcomes analyzed included duration of dialysis requirement (hours), incidence of AKI, and posttransplantation creatinine clearance. AKI was defined as per the Acute Kidney Injury Network criteria [30]. We also abstracted data regarding adverse effects of natriuretic peptides such as hypotension and arrhythmias. 

### 3.1. Data Analysis and Quantitative Data Synthesis

We analyzed data as per guidelines in the Cochrane Reviewers' Handbook [31]. All the analyses were performed using RevMan 4.2.10 (Cochrane Collaboration, Oxford, UK). Dichotomous data outcomes from individual studies were analyzed according to the Mantel-Haenszel model to compute individual odds ratio (OR) with 95% confidence intervals (CI). Where continuous scales of measurement were used to assess the effects of treatment, the weighted mean difference (WMD) was used. Treatment effects were pooled with the fixed-effects model. Statistical significance was set at the 2-tailed 0.05 level for hypothesis testing. Statistical heterogeneity was analyzed using *I* ^2^ test [32]. *I* ^2^ values of 25%, 50%, and 75% correspond to low, medium and high levels of statistical heterogeneity. We constructed funnel plots to explore publication bias. 

### 3.2. Sensitivity Analyses

Sensitivity analyses were conducted by switching from fixed-effect to random-effect models and by computing relative risks. We also planned to repeat the analyses (if adequate number of studies were to be available) by restricting it to patients undergoing nonrenal solid organ transplantation, restricting to high quality studies, and restricting to studies that included participants with preexisting renal impairment.

## 4. Results

Database searches and snowballing yielded a total of 123 citations. Excluding 98 nonrelevant titles and abstracts, we retrieved 25 studies in complete form and assessed them according to the selection criteria. A total of 18 studies were further excluded, since they involved evaluation in nonsolid organ transplant setting. Our analysis finally identified 7 eligible studies comprising total 238 participants (118 natriuretic peptide group; 120 control group) [4, 6, 22–26]. Characteristics of the included studies are summarized in Table 1. Mean age of the participants was 44 years and 40% participants were females. Four studies (135 participants) evaluated the role of human atrial natriuretic peptide [4, 6, 23, 26]. Three studies (103 participants) evaluated the role of urodilatin [22, 24, 25]. No eligible studies were identified that involved administration of brain natriuretic peptide or C-type natriuretic peptide. Natriuretic peptides were generally given via intravenous infusion route, and one study included administration in renal allograft renal artery followed by intravenous infusion [23]. The dosages of natriuretic peptides varied widely amongst the studies; human natriuretic peptide was typically administered at dosages from 0.0125 *μ*g/kg/min to 0.05 *μ*g/kg/min, and urodilatin was administered at dose of 20 ng/kg/min or 40 ng/kg/min. The durations of natriuretic peptide administration also varied widely amongst the studies from anywhere between 4 hours to 7 days. Control intervention was placebo in all studies except in one where it was furosemide infusion with potassium canrenoate [4].

Solid organ transplantation surgeries included liver transplantation [4, 24, 25], renal transplantation [6, 23, 26], and heart transplantation [22]. None of the studies were conducted in the setting of combined solid organ transplantation or in lung transplantation. Four studies were designed to assess the effects of natriuretic peptides in patients with preexisting impaired renal function [6, 23, 24, 26]. Natriuretic peptide administration was started either at or immediately after the surgery in all studies. None of the studies except one [4] had no standardized criteria for initiation of dialysis, and this decision was largely left to the treating clinicians in the remaining studies.

Jadad scores for the included studies are outlined in Table 1. The overall quality of the included studies was suboptimal with only 2 studies being of high quality [22, 26]. In studies with moderate and low quality, descriptions of randomization and blinding methods were poorly reported [4, 6, 23–25]. All the included studies had single center enrollment of patients, and none acknowledged support from the pharmaceutical industry.

### 4.1. Primary Outcomes

**Figure 1:**
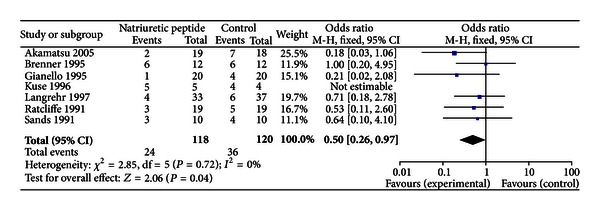


Data on AKI requiring dialysis were reported in all 7 studies. Pooled estimate showed that the use of natriuretic peptide was associated with reduction in AKI requiring dialysis (OR 0.50 [0.26–0.97], *I* ^2^ = 0%) (Figure 1). None of the studies reported any 30-day or in-hospital mortality events; hence, meta-analyses could not be conducted for this outcome.

### 4.2. Secondary Outcomes and Adverse Effects

Only one study reported duration of dialysis requirement and in this study use of natriuretic peptide was associated with a significant reduction in the duration of dialysis requirement (WMD −44.0 hours, [−60.5 to −27.5 hours]) [22]. Sufficient data were not available from the individual RCTs to compute the AKI incidence as defined by the Acute Kidney Injury Network criteria; hence this outcome could not be analyzed. Two studies reported data on postsurgery creatinine clearance [6, 26]. Pooled analyses for this outcome showed a nonstatistically significant trend towards improvement in creatinine clearance in participants that received natriuretic peptides (WMD 5.5 mL/min, [−1.3 to 12.2 mL/min]). 

We analyzed adverse effect profile of natriuretic peptide as reported in individual studies. None of the studies reported any adverse events such as hypotension or arrhythmias in either arm of the RCTs.

### 4.3. Sensitivity Analyses

Sensitivity analyses were performed by switching from random-effect to fixed-effect models, and by computing relative risks. These analyses did not change the overall results for all the outcomes.

Further sensitivity analyses as originally proposed by restricting to nonrenal solid organ transplant settings, restricting to studies with participants that have preexisting renal impairment prior to the initiation of intervention, and restricting to high quality studies were not conducted due to highly limited number of small studies that were available to conduct meta-analyses.

**Figure 2:**
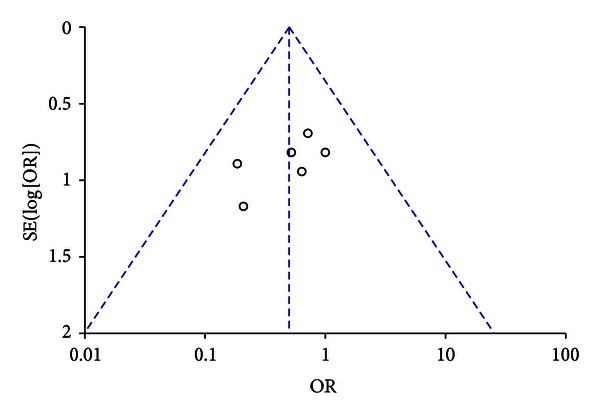


Assessment of validity and robustness of these findings by means of a funnel plot suggested possibility of small study publication bias (Figure 2).

## 5. Discussion

AKI following a solid organ transplantation is a major cause of morbidity and mortality [10]. Unfortunately, no effective interventions are available to prevent or treat this condition, and thus there is an urgent need for development of new agents. Multiple factors, including renal hypoperfusion, hypovolemia, ischemia-reperfusion, neurohumoral including renin-angiotensin system activation, and nephrotoxin exposure especially anti-rejection medications, are known to contribute to this renal dysfunction in organ transplantation setting [4, 10]. The use of natriuretic peptides with their properties noted in animal models such as vasorelaxation, natriuresis, diuresis, and aldosterone inhibition appears to be a potentially effective option to manage cardiovascular surgery associated renal dysfunction [4, 13–17].

Our meta-analysis assessed the efficacy for renal outcomes and safety of natriuretic peptides in patients undergoing solid organ transplantation. Our comprehensive literature review found that most studies addressing this topic are small and lack the power to reach statistical significance on their own for clinically meaningful outcomes (such as dialysis and mortality). However, pooled analysis of the current available evidence shows that the administration of natriuretic peptides is associated with reduction in the postsurgery dialysis requirement along with a possible reduction in postsurgery dialysis duration and a nonstatistically significant trend towards improvement in creatinine clearance in participants that received natriuretic peptides. In this review, natriuretic peptides were well tolerated with no reports of hypotension and arrhythmias.

Larger and adequately powered studies designed to evaluate atrial natriuretic peptide in other settings, such as acute tubular necrosis from conditions such as sepsis, have been negative [33, 34]. Dose of atrial natriuretic peptide preparations administered in these studies was much larger (up to 0.20 *μ*g/kg/minute) and was associated with significantly higher incidence of hypotension [14–17]. When renal perfusion pressure falls below 100 mm Hg, the renal blood flow in the cortex and medulla decreases in response, and that in the medulla is poorly autoregulated [35]. Under this condition, atrial natriuretic peptide induced hypotension could potentially negate the beneficial effects [14–17]. By contrast, studies performed in solid organ transplantation setting in our review administered lower doses of atrial natriuretic peptide preparations and were not associated with significant increase in adverse events. This differential risk benefit ratio associated with dosing of natriuretic peptides should be taken into consideration while planning further RCTs. Future studies should also systematically collect data on urine output and serum creatinine to compute incidence of AKI as defined by the Acute Kidney Injury Network criteria [30].

Our systematic review has limitations, similar to our prior work that analyzed effects of natriuretic peptides in other settings such as cardiovascular surgery [14–17, 31]. The outcomes considered in our review were not necessarily the primary outcomes of interest to the study authors, and hence the included studies were underpowered to detect any significant difference for outcomes such as AKI requiring dialysis. There were no uniform indications for dialysis in most of the included trials and the decision to initiate dialysis was left to the participating physicians. This may have introduced potential confounding for AKI requiring dialysis outcome analysis. Additionally, most included studies were conducted prior to the year 2000, and considerable differences in pathophysiology as well as epidemiology of AKI compared to the recent years are possible. Due to limited number of small studies, we could not conduct the pre-specified sensitivity analyses that may have addressed the heterogeneity introduced by different surgical procedures. Overall suboptimal quality and inadequate power of the included studies limits power of our meta-analysis and necessitates confirmation of these findings by future better-conducted and adequately powered studies. We also did not have information from the included studies on pretransplant variables that may impact renal outcomes. Despite rigorous search strategy, our funnel plot analyses suggested possibility of small study publication bias. Another limitation of our review is that information on pre-specified outcome measures was not available in all studies despite contacting the original authors. Despite these limitations, our review identifies natriuretic peptides as an intervention, that is well tolerated and possibly effective in preventing dialysis requiring AKI, that is commonly associated with solid organ transplantation. Our review prompts further randomized controlled trials of this intervention. 

In conclusion, thecurrent literature analyzing studies evaluating administration of natriuretic peptides in solid organ transplantation setting may be associated with significant improvements in renal outcomes. Given the limitations of meta-analysis, these observations need to be confirmed in a larger, adequately powered, prospective multicenter study.

## Figures and Tables

**Table 1:** Characteristics of included randomized controlled trials.

Study, year	Country	Setting	Intervention	Patients randomized		Mean age, yrs		Baseline renal function ∞		Reported outcomes	****Jadad ****score
				NP	Control	NP	Control	NP	Control		
Akamatsu et al., 2005 [4]	Japan	Live donor liver transplantation (recipients with model for end-stage liver disease scores >15)	Synthetic hANP infusion 0.05 to 0.1 *μ*g/kg/min for 5 days	19	18	48	51	48 ± 22	51 ± 18	AKI requiring RRT, mortality, adverse events	3

Brenner et al., 1995 [22]	Germany	Heart transplantation	Urodilatin 40 ng/kg/min for 6 days	12	12	NR		147 ± 30	102 ± 19	AKI requiring RRT, duration of RRT, mortality, adverse events	5

Gianello et al., 1995 [23]	Denmark	Cadaveric renal transplantation	Synthetic hANP 100 *μ*g bolus into renal allograft artery followed by infusion at 0.01 to 0.03 *μ*g/kg/min until serum creatinine <2 mg/dL	20	20	36	38	NR		AKI requiring RRT, mortality, adverse events	4

Kuse et al., 1996 [24]	Germany	Liver transplantation (recipients with emerging ARF with criteria such as refractory anuria/oliguria <0.5 mL/kg/hr, increase in serum creatinine ≥200% or BUN ≥25 mmol/L)	Urodilatin at 20 ng/kg/min for 7 hours	5	4	49	44	2.0 ± 1.4	2.3 ± 1.1	AKI requiring RRT, mortality, adverse events	4

Langrehr et al., 1997 [25]	Denmark	Liver transplantation	Urodilatin at 20 ng/kg/min for 7 days	33	37	44	47	1.2 ± 0.1	1.0 ± 0.1	AKI requiring RRT, mortality, adverse events	2

Ratcliffe et al., 1991 [26]	United States	Cadaveric renal transplantation	Atriopeptin 0.0125 to 0.1 *μ*g/kg/min for 12 hours	19	19	NR	NR	1.1 ± 0.4	1.0 ± 0.4	AKI requiring RRT, mortality, posttransplant CrCl, adverse events	5

Sands et al., 1990 [6]	United States	Cadaveric renal transplantation	Synthetic hANP 50 *μ*g bolus followed by 0.1 *μ*g/kg/min for 4 hours	10	10	44	41	NR		AKI requiring RRT, mortality, posttransplant CrCl, adverse events	3

## References

[1] Rosner MH, Okusa MD (2006). Acute kidney injury associated with cardiac surgery. *Clinical Journal of the American Society of Nephrology*.

[2] Thakar CV, Christianson A, Freyberg R, Almenoff P, Render ML (2009). Incidence and outcomes of acute kidney injury in intensive care units: a veterans administration study. *Critical Care Medicine*.

[3] Siew ED, Deger SM (2012). Recent advances in acute kidney injury epidemiology. *Current Opinion in Nephrology and Hypertension*.

[4] Akamatsu N, Sugawara Y, Tamura S (2005). Prevention of renal impairment by continuous infusion of human atrial natriuretic peptide after liver transplantation. *Transplantation*.

[5] Boyle JM, Moualla S, Arrigain S (2006). Risks and outcomes of acute kidney injury requiring dialysis after cardiac transplantation. *The American Journal of Kidney Diseases*.

[6] Sands JM, Neylan JF, Olson RA, O’Brien DP, Whelchel JD, Mitch WE (1990). Atrial natriuretic factor does not improve the outcome of cadaveric renal transplantation. *Journal of the American Society of Nephrology*.

[7] Pham PTT, Slavov C, Pham PCT (2009). Acute kidney injury after liver, heart, and lung transplants: dialysis modality, predictors of renal function recovery, and impact on survival. *Advances in Chronic Kidney Disease*.

[8] Rocha PN, Rocha AT, Palmer SM, Davis RD, Smith SR (2005). Acute renal failure after lung transplantation: incidence, predictors and impact on perioperative morbidity and mortality. *The American Journal of Transplantation*.

[9] Ishani A, Erturk S, Hertz MI, Matas AJ, Savik K, Rosenberg ME (2002). Predictors of renal function following lung or heart-lung transplantation. *Kidney International*.

[10] Clajus C, Hanke N, Gottlieb J (2012). Renal comorbidity after solid organ and stem cell transplantation. *The American Journal of Transplantation*.

[11] Cabezuelo JB, Ramirez P, Acosta F (2002). Prognostic factors of early acute renal failure in liver transplantation. *Transplantation Proceedings*.

[12] Siedlecki A, Irish W, Brennan DC (2011). Delayed graft function in the kidney transplant. *The American Journal of Transplantation*.

[13] Vesely DL (2003). Natriuretic peptides and acute renal failure. *The American Journal of Physiology*.

[14] Nigwekar SU, Hix JK (2009). The role of natriuretic peptide administration in cardiovascular surgery-associated renal dysfunction: a systematic review and meta-analysis of randomized controlled trials. *Journal of Cardiothoracic and Vascular Anesthesia*.

[15] Nigwekar SU, Navaneethan SD, Parikh CR, Hix JK (2009). Atrial natriuretic peptide for preventing and treating acute kidney injury. *Cochrane Database of Systematic Reviews*.

[16] Nigwekar SU, Navaneethan SD, Parikh CR, Hix JK (2009). Atrial natriuretic peptide for management of acute kidney injury: a systematic review and meta-analysis. *Clinical Journal of the American Society of Nephrology*.

[17] Nigwekar SU, Waikar SS (2011). Diuretics in acute kidney injury. *Seminars in Nephrology*.

[18] Opgenorth TJ, Novosad EI (1990). Atrial natriuretic factor and endothelin interactions in control of vascular tone. *European Journal of Pharmacology*.

[19] Sagnella GA (2000). Practical implications of current natriuretic peptide research. *Journal of the Renin-Angiotensin-Aldosterone System*.

[20] Gagelmann M, Hock D, Forssmann WG (1988). Urodilatin (CDD/ANP-95-126) is not biologically inactivated by a peptidase from dog kidney cortex membranes in contrast to atrial natriuretic peptide/cardiodilatin (*α*-hANP/CDD-99-126). *FEBS Letters*.

[21] Lainchbury JG, Burnett JC, Meyer D, Redfield MM (2000). Effects of natriuretic peptides on load and myocardial function in normal and heart failure dogs. *The American Journal of Physiology*.

[22] Brenner P, Meyer M, Reichenspurner H (1995). Significance of prophylactic urodilatin (INN: ularitide) infusion for the prevention of acute renal failure in patients after heart transplantation. *European Journal of Medical Research*.

[23] Gianello P, Carlier M, Jamart J (1995). Effect of 1-28*α*-h atrial natriuretic peptide on acute renal failure in cadaveric renal transplantation. *Clinical Transplantation*.

[24] Kuse ER, Meyer M, Constantin R (1996). Urodilatin (INN: ularitide). A novel peptide for the treatment of postoperative acute renal failure following liver transplantation. *Anaesthesist*.

[25] Langrehr JM, Kahl A, Meyer M (1997). Prophylactic use of low-dose urodilatin for prevention of renal impairment following liver transplantation: a randomized placebo-controlled study. *Clinical Transplantation*.

[26] Ratcliffe PJ, Richardson AJ, Kirby JE, Moyses C, Shelton JR, Morris PJ (1991). Effect of intravenous infusion of atriopeptin 3 on immediate renal allograft function. *Kidney International*.

[27] Kentsch M, Drummer C, Gerzer R, Muller-Esch G (1995). Severe hypotension and bradycardia after continuous intravenous infusion of urodilatin (ANP 95–126) in a patient with congestive heart failure. *European Journal of Clinical Investigation*.

[28] Moher D, Cook DJ, Eastwood S, Olkin I, Rennie D, Stroup DF (1999). Improving the quality of reports of meta-analyses of randomised controlled trials: the QUOROM statement. *The Lancet*.

[29] Jadad AR, Moore RA, Carroll D (1996). Assessing the quality of reports of randomized clinical trials: is blinding necessary?. *Controlled Clinical Trials*.

[30] Mehta RL, Kellum JA, Shah SV (2007). Acute Kidney Injury Network: report of an initiative to improve outcomes in acute kidney injury. *Critical Care*.

[31] Alderson PGS, Higgins JPT (2004). *Analysing and Presenting Results*.

[32] Higgins JPT, Thompson SG, Deeks JJ, Altman DG (2003). Measuring inconsistency in meta-analyses. *The British Medical Journal*.

[33] Lewis J, Salem MM, Chertow GM (2000). Atrial natriuretic factor in oliguric acute renal failure. *The American Journal of Kidney Diseases*.

[34] Allgren RL (1998). Update on clinical trials with atrial natriuretic peptide in acute tubular necrosis. *Renal Failure*.

[35] Mattson DL, Lu S, Roman RJ, Cowley AW (1993). Relationship between renal perfusion pressure and blood flow in different regions of the kidney. *The American Journal of Physiology*.

